# Simultaneous synthesis of perfusion and ventilation images from CT using a dual‐decoder residual attention network for lung disease diagnosis

**DOI:** 10.1002/acm2.70498

**Published:** 2026-02-26

**Authors:** Meng Wang, Xi Liu, Haoze Li, Meixin Zhao, Tianyu Xiong, David Huang, Jing Cai, Weifang Zhang, Li‐Sheng Geng, Ruijie Yang

**Affiliations:** ^1^ Department of Nuclear Medicine Peking University Third Hospital Beijing China; ^2^ School of Physics Beihang University Beijing China; ^3^ Department of Radiation Oncology Cancer Center Peking University Third Hospital Beijing China; ^4^ Department of Health Technology and Informatics The Hong Kong Polytechnic University Hong Kong China; ^5^ Peng Huanwu Collaborative Center for Research and Education Beihang University Beijing China; ^6^ Medical Physics Graduate Program Duke Kunshan University Kunshan China; ^7^ Beijing Key Laboratory of Advanced Nuclear Materials and Physics Beihang University Beijing China

**Keywords:** cross modality, deep learning, image synthesis, lung functional imaging

## Abstract

**Background:**

Deep learning algorithms can synthesize pulmonary functional images from CT images. However, previous studies have only been able to predict either ventilation or perfusion from CT, limiting the holistic evaluation of lung function.

**Purpose:**

This study aimed to develop a deep learning‐based framework for simultaneously generating lung perfusion and ventilation images from three‐dimensional CT.

**Methods:**

A total of 98 cases who underwent single‐photon emission CT perfusion images (SPECT PI) with ^99m^Tc‐labeled macroaggregated albumin, ventilation images (VI) with ^99m^Tc‐Technegas, and three‐dimensional CT images were collected. The three‐dimensional CT and SPECT images were registered and cropped to include only the lung parenchyma. A dual‐decoder residual attention network (DDRAN) was constructed to generate both PI and VI simultaneously from three‐dimensional CT images. For comparative assessment, we additionally employed a conventional single‐decoder residual attention network (RAN) to individually generate PI and VI. The structural similarity index (SSIM) and Spearman's rank correlation coefficient (Rs) were utilized to assess voxel‐wise agreement. Additionally, the Dice similarity coefficient (DSC) was applied to evaluate function‐wise concordance. We used the Wilcoxon signed‐rank test to statistically evaluate the differences between the images synthesized by DDRAN and RAN. Beyond image‐similarity metrics, we evaluated overall model performance using threshold‐based classification. Lastly, a two‐part reader study was conducted: (I) qualitative image acceptability for clinical review, and (II) illustrative diagnostic interpretation based on synthesized image pairs alone.

**Results:**

Overall, DDRAN and RAN achieved comparable performance. The average SSIM values of the DDRAN/RAN model were 0.871/0.866 (*p* < 0.05) for PI and 0.830/0.825 (*p* < 0.05) for VI, and the Rs values were 0.836/0.819 and 0.732/0.731, respectively. The DDRAN/RAN model achieved average DSC values of 0.795/0.796 for PI and 0.708/0.718 for VI in low‐function regions, and 0.857/0.849 for PI and 0.793/0.793 for VI in high‐function regions. In the two‐part reader study, the synthesized perfusion and ventilation images received almost acceptable scores across all experience levels and demonstrated diagnostic potential.

**Conclusions:**

We have developed a dual‐decoder residual attention network that enables the simultaneous synthesis of lung perfusion and ventilation images from three‐dimensional CT. Preliminary results indicate moderate‐to‐high structural‐wise and functional‐wise concordances, and our proposed model demonstrates comparable accuracy when benchmarked against single‐decoder models. The synthesized perfusion and ventilation images can potentially be used for precise diagnosis and guiding functional lung avoidance radiotherapy.

## 1 INTRODUCTION

Pulmonary functional imaging is a key modality to aid in the diagnosis of lung diseases such as pulmonary embolism (PE), chronic obstructive pulmonary disease (COPD), and pulmonary hypertension.^1^, ^2^ It also plays a crucial role in guiding functional lung avoidance radiation therapy (FLART) to improve patient prognosis.^3^, ^4^, ^5^, ^6^ Radiation‐induced lung injury (RILI) is a common complication in lung cancer patients undergoing thoracic radiotherapy.^7^, ^8^ Studies report that the incidence of Grade ≥2 radiation pneumonitis after radiotherapy is approximately 28%,^9^ with fatal pneumonitis occurring in about 2% of cases.^10^, ^11^ RILI has become a major factor limiting radiation dose escalation, compromising local tumor control, and significantly impacting the quality of life and survival of lung cancer patients after radiotherapy.^11^, ^12^, ^13^

Pulmonary functional images typically comprise ventilation images, which assess airflow, and perfusion images, which assess blood flow in the lungs.^14^ Nuclear medicine imaging is currently the gold standard for obtaining such images. This involves administering radioisotopes (via injection or inhalation) and scanning the region of interest. The most commonly used nuclear medicine modalities are single‐photon emission computed tomography (SPECT)^15^, ^16^, ^17^ and positron emission tomography (PET).^18^, ^19^ With advancements in imaging technology, functional information can also be obtained through magnetic resonance imaging (MRI)^20^, ^21^ and contrast‐enhanced dual‐energy computed tomography (DECT).^22^ While these methods offer accurate functional information, these techniques entail extra radiation exposure and additional costs.^23^

Computed tomography (CT) can also provide lung ventilation information. Ventilation images can be generated by analyzing the respiration‐induced changes in air volume within the lungs using 4D CT images.^24^, ^25^ Alternatively, parametric response maps generated by registering inspiratory and expiratory CT scans can serve as functional substitutes, highlighting areas of air trapping and aiding in the diagnosis of small airway diseases.^25^, ^26^ While this approach is highly accessible and involves no additional radiation, 4D CT‐based lung ventilation imaging relies heavily on the accuracy of deformable registration algorithms, which may compromise its computational precision and robustness.^27^, ^28^

In recent years, extensive research has been conducted on applying deep learning (DL) algorithms, especially convolutional neural network (CNN),^29^ to diverse medical imaging tasks, encompassing diagnosis,^30^ segmentation,^31^ image synthesis,^32^, ^33^ treatment planning,^34^ and quality assurance.^35^ CNNs are DL algorithms primarily composed of convolutional, pooling, and fully connected layers.^29^ Several studies^36^, ^37^, ^38^, ^39^, ^40^, ^41^ have applied CNNs for generating lung functional images due to their powerful feature extraction capabilities. For instance, Jiang et al.^36^ proposed a conditional generative adversarial network to obtain two‐dimensional (2D) perfusion SPECT images from 2D CT. Ren et al.^37^, ^38^ constructed an attention residual neural network to directly synthesize three‐dimensional (3D) perfusion SPECT images from 3D CT, achieving moderate‐to‐high voxel‐wise accuracy. Liu et al.^40^ first applied a DL model to generate ventilation SPECT images from 4D CT. Similarly, Kajikawa et al.^39^ utilized a U‐Net to generate ventilation images only from 3D CT and evaluated its accuracy. These studies confirm that CT images contain latent information that DL algorithms can extract for synthesizing pulmonary functional images. However, prior studies were limited to synthesizing only a single functional modality (ventilation or perfusion) from the input CT images. This is insufficient for a comprehensive evaluation of lung function and precise lung disease diagnosis. For example, PE is characterized by normal ventilation with abnormal perfusion, whereas COPD typically presents with normal perfusion but abnormal ventilation. Accurate diagnosis often requires both perfusion and ventilation images to differentiate disease types. However, acquiring SPECT images requires a relatively long acquisition time, and the lung perfusion and ventilation images must be obtained separately, resulting in doubled radiation exposure for patients. Consequently, it is worthwhile to investigate how to rapidly obtain both perfusion and ventilation images with lower radiation doses, especially for emergency patients.

To solve the issues mentioned above, we developed a 3D dual‐decoder residual attention network (DDRAN) to simultaneously generate DDRAN‐based perfusion images (DDRAN‐PI) and ventilation images (DDRAN‐VI) from 3D CT. We used Spearman's rank correlation coefficient (Rs) and the structural similarity index (SSIM) to evaluate the structural similarity between the synthesized images and the ground truth. Meanwhile, we utilized the Dice similarity coefficient (DSC) to evaluate the functional concordance of the synthesized images, laying a foundation for its application in FLART. Furthermore, a two‐part reader study was conducted to validate its clinical feasibility. Our study primarily contributes to the following aspects:

1. We proposed a specially tailored dual decoder DL framework that simultaneously synthesized co‐registered perfusion and ventilation SPECT‐like maps from 3D CT, thus enabling paired functional assessment without the need for acquiring two separate SPECT scans.
2. We showed that the proposed dual‐decoder design achieved comparable image‐ and region‐level agreement with two separately trained single‐output baselines, while providing practical workflow advantages (one model, shared encoder, and consistent voxel‐wise pairing).
3. We provided preliminary reader‐based evidence on perceptual image acceptability and qualitative diagnosis when only synthesized functional images were available, further demonstrating the method's clinical value and translational potential.

## 2 MATERIALS AND METHODS

### 2.1 Data acquisition and preprocessing

#### 2.1.1 Patients and image acquisition

Ninety‐eight patients who underwent ventilation/perfusion (V/Q) SPECT/CT scanning at our hospital were included in this study. Among them, 72 were diagnosed with one of the following diseases: COPD, PE, lung cancer, or pneumonia, and the remaining 26 were normal cases. Patient characteristics are summarized in Table 1. The SPECT/CT acquisition protocol is detailed below.

**TABLE 1:** Clinical characteristics of the patients.

Characteristics		Number	Percent
Gender	Male	31	31.63%
	Female	67	68.37%
Age	Mean ± SD (Year)	65.79 ± 17.05	–
Diagnosis	COPD	28	28.57%
	PE	23	23.47%
	Lung cancer	4	4.08%
	Pneumonia	17	17.35%
	Normal case	26	26.53%

Abbreviation: COPD, chronic obstructive pulmonary disease; PE, pulmonary embolism; SD, standard deviation.

V/Q SPECT/CT scanning was conducted over two days with a dual‐head SPECT/CT instrument (Symbia Intevo T16 SPECT‐CT, Siemens, Germany) equipped with low‐energy, high‐resolution collimators (window center/width in keV/%: 140/20). Perfusion assessments were performed on day one. Participants were placed in a supine position and intravenously injected with 111 MBq of ^99m^Tc‐macroaggregated albumin, and then SPECT perfusion imaging was performed with a 128 × 128 matrix, zoom 1.0, and 32 views per head over 180°, and the duration of each view was 20s. Ventilation assessments were conducted on day two. Participants inhaled 20–30 MBq ^99m^Tc‐Technegas from a Technegas generator (Vita Medical Limited, Sydney, Australia), and then ventilation SPECT imaging was conducted via the same methods described for perfusion imaging. SPECT perfusion and ventilation datasets were reconstructed via ordered‐subset expectation maximization with eight subsets and two iterations. Chest CT was performed during free‐breathing with the following parameters: pitch 0.95, rotation time 0.6 s, quality reference mAs 30 (with CARE Dose4D tube current modulation, Siemens Healthineers), and tube voltage 130 kV. Filtered back projection was used for CT reconstruction. CT image reconstruction was performed with commonly used parameters for slice thickness (2.5 mm) and increments (1.25 mm).

#### 2.1.2 CT and V/Q SPECT preprocessing

First, each pulmonary V/Q image was registered to the corresponding CT image using MIM Software (v.6.3.4, MIM Software Inc., Cleveland, OH, USA). Then, we utilized the TotalSegmentator tool proposed by Wasserthal et al.^42^ to segment the left and right lungs, enabling the model to focus on pulmonary regions. TotalSegmentator was constructed based on nnUNet,^43^ trained on numerous CT images, and had stable performance in common segmentation scenarios. Next, the images were cropped into the dimension of 64×128×128 pixels to accelerate the training process and reduce the computational cost.

Contrast enhancement is a method of expanding the contrast of relevant features, and histogram equalization (HE) can make the histogram of the CT images uniform to obtain an optimal overall contrast.^44^, ^45^ We equalized the CT images within the HU range of −1000 to −300^37^ as shown in Equation (1):

(1) $$\begin{array}{c} CT{\left(x,y,z\right)}^{\prime }=\frac{chf\left[CT\left(x,y,z\right)\right]-chf\left[-1000\right]}{chf\left[-300\right]-chf\left[-1000\right]}\;\; \end{array}$$

where the cumulative histogram function (chf) indicates the cumulative counts of pixels per HU value. After HE processing, each pixel value of the CT images is between 0 and 1.

On the other hand, voxel values in SPECT images are relative and may vary depending on patients’ respiratory capacity, breathing frequency, and lung function. Therefore, SPECT images are usually normalized to a percentile value instead of a fixed value.^27^, ^46^, ^47^ In this study, we normalized the SPECT images to the 75th percentile value and clipped pixel values exceeding 1 to 1, as in previous studies^37^, ^39^ as defined in Equation (2):

(2) $$\begin{array}{c} SPECT{\left(x,y,z\right)}^{\prime }=\min\left\{1,\frac{SPECT\left(x,y,z\right)}{SPECT\left(75th\;percentile\right)}\right\}\;\; \end{array}$$

### 2.2 DL method for V/Q SPECT generation

#### 2.2.1 DL architecture and training

It has been shown that a multi‐decoder network performs well in automatic segmentation.^48^, ^49^ This architecture reduces overfitting by employing a shared encoder and is also more memory‐efficient. Thus, we adopted a residual attention U‐Net‐based dual‐decoder CNN model for our lung functional image synthesis task.^50^, ^51^

**FIGURE 1:**
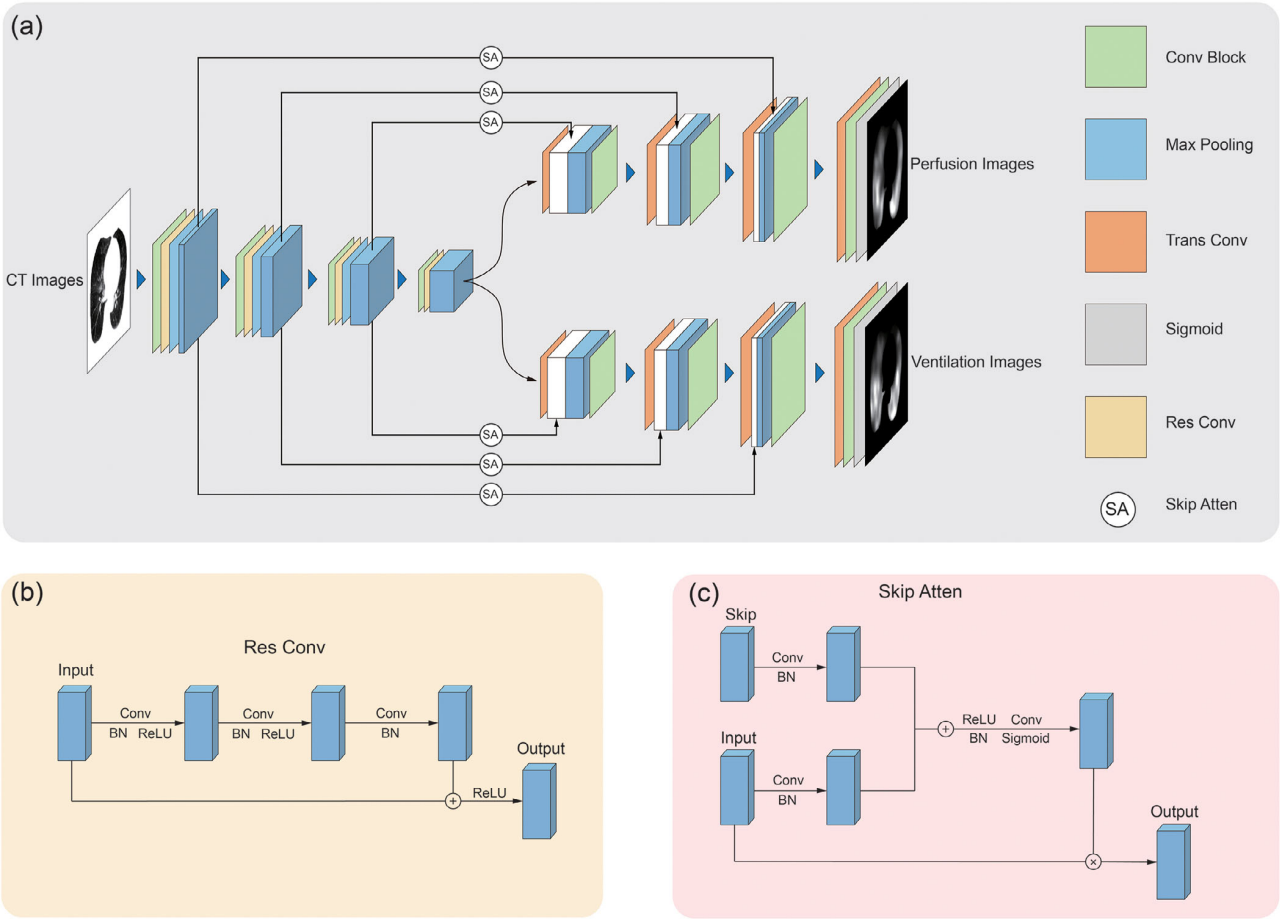
(a) The architecture of our dual‐decoder residual attention network (DDRAN). The green and orange boxes represent the convolution and transpose convolution blocks, respectively; the blue box represents max pooling layer, the yellow box represents the residual convolution module, the gray box represents the sigmoid function, and SA denotes the skip attention module. (b) Details of the residual convolution module. (c) Details of the skip attention module.

Figure 1(a) shows the architecture of our DL model. This model comprises a common encoder and two distinct decoders used to synthesize perfusion and ventilation images, respectively. The encoding path consists of four convolution blocks, four residual blocks for feature extraction, and three 2×2×2 max‐pooling layers with a stride of 2 for down‐sampling. Each convolution block consists of repeated modules, containing a 3×3×3 convolution layer with a kernel size of 3×3×3, a stride of 1, a padding of 1, a rectified linear unit (ReLU), and a batch normalization layer. As shown in Figure 1(b), the residual blocks are used to deepen the network and improve training efficiency, as in ResNet.^51^ Both decoders share the same structure. Each decoder path consists of three transpose convolution blocks for up‐sampling and three convolution blocks to reduce the number of channels. At each image resolution stage, a skip attention module, shown in Figure 1(c), is employed to concatenate the feature maps from the encoder blocks with those from the corresponding decoder blocks,^50^ enabling the network to selectively focus on salient features of perfusion and ventilation images, respectively. Following this, transpose and convolution blocks are used to restore the images' original size and reduce the number of channels to 1. At the final layer, a sigmoid function processes the outputs of the previous layers and maps output values to the range (0,1).

We randomly divided the dataset into three sub‐datasets with a ratio of 6:2:2. Ultimately, 62 cases were utilized for model training, 18 cases were designated as the validation set, and the remaining 18 cases were reserved as the test set to independently assess the model's performance on unseen data. We employed the mean absolute error (MAE) as the loss function, calculating separate losses between each generated image (perfusion and ventilation) and its corresponding ground truth. The total loss function is then defined as the sum of these two MAE losses, as shown in Equation (3):

(3) $$Loss=\frac{1}{N}\sum_{i=1}^{N}\left|y_{i}^{per}-p_{i}^{per}\right|+\frac{1}{N}\sum_{i=1}^{N}\left|y_{i}^{ven}-p_{i}^{ven}\right|\;\;$$

where $y_{i}^{per}$, $y_{i}^{ven}$, $p_{i}^{per}$ and $p_{i}^{ven}$ are the predicted and ground truth of perfusion and ventilation values at voxel i, respectively. An Adam optimizer ($l_{r}=0.001$, $\beta_{1}=0.9$, $\beta_{2}=0.999$, $\varepsilon =1.0\times 10^{-8}$) was utilized to optimize the parameters. The maximum training epoch was set to 100, and the batch size was set to 4. To mitigate overfitting, we selected the model that performed best on the validation set and evaluated its performance on the independent test set. All experiments were performed using a NIVIDA A800 GPU with 80GB memory.

#### 2.2.2 Quantitative evaluation

We used Rs and the SSIM to evaluate the performance of the proposed model. Rs is used to evaluate the intensity correlation between the predicted and SPECT images, with 1 indicating a perfect positive correlation, −1 indicating a perfect negative correlation, and 0 indicating no correlation. Rs is defined as follows in Equation (4):

(4) $$Rs=\frac{\sum_{i=1}^{N}\left[\left(y_{i}-\bar{y}\right)\cdot \left(p_{i}-\bar{p}\right)\right]}{\sqrt{\sum_{i=1}^{N}{\left(y_{i}-\bar{y}\right)}^{2}}\sqrt{\sum_{i=1}^{N}{\left(p_{i}-\bar{p}\right)}^{2}}}\;\;$$

where $y_{i}$, $p_{i}$, $\bar{y}$, and $\bar{p}$ denote the value and the average value at voxel i for the SPECT images y and predicted images p, respectively. N denotes the total number of voxels within the lung parenchyma area.

SSIM is used to evaluate the perceptual similarity between two images. It considers not only the differences in pixel values but also factors such as brightness, contrast, and structure and can better assess the overall quality and similarity of images comprehensively. SSIM values range from 0 to 1, where 1 means that the two images are identical and 0 indicates that they are completely different. SSIM is defined as follows in Equation (5):

(5) $$\begin{array}{cc} SSIM & =L\left(y,p\right)\cdot C\left(y,p\right)\cdot S\left(y,p\right)=\frac{2\mu_{y}\mu_{p}+C_{1}}{\mu_{y}^{2}+\mu_{p}^{2}+C_{1}}\\ & \;\cdot \frac{2\sigma_{y}\sigma_{p}+C_{2}}{\sigma_{y}^{2}+\sigma_{p}^{2}+C_{2}}\cdot \frac{2\sigma_{yp}+C_{2}}{2\sigma_{y}\sigma_{p}+C_{2}}\;\; \end{array}$$

where L(y,p), C(y,p) and S(y,p) represent the luminance comparison value, contrast comparison value and structure comparison value between the SPECT images y and the predicted images p, respectively, $\mu_{y}$, $\mu_{p}$, $\sigma_{y}$, $\sigma_{p}$ and $\sigma_{yp}$ are the local means, standard deviations and covariance between y and p. $C_{1}={\left(k_{1}L\right)}^{2}\;$ and $C_{2}={\left(k_{2}L\right)}^{2}\;$ are two small constants to prevent division by zero, where L represents the dynamic range of pixel values, $k_{1}$ and $k_{2}$ are typically set to 0.01 and 0.03, respectively.

Furthermore, we used the DSC to evaluate the functional consistency between the V/Q SPECT and predicted images. Following previous studies,^52^, ^53^ pixel values greater than 0.66 were categorized as high‐function lung (HFL) volumes, and those below 0.66 were categorized as low‐function lung (LFL) volumes. DSC is defined as follows in Equation (6):

(6) $$DSC=\frac{2\left|p\cap y\right|}{\left|p\right|+\left|y\right|}\;\;$$

where p and y are the high‐ and low‐function lung volumes of the predicted and SPECT images, respectively. Concurrently, we binarized the images using the same threshold as in the DSC calculation, labeling high‐function regions as 1 and low‐function regions as 0, and then we computed true positive rate (TPR), true negative rate (TNR), false positive rate (FPR), and false negative rate (FNR) to quantify the proportion of misclassified regions in the synthesized functional images. Accuracy, precision, and F1 score were also reported to evaluate model performance.

For a more comprehensive comparative evaluation, we employed two conventional single‐decoder residual attention networks (RANs) to independently generate perfusion images (RAN‐PI) and ventilation images (RAN‐VI). Subsequently, we utilized the Wilcoxon signed‐rank test to statistically assess the differences between the images predicted by our proposed DDRAN and those predicted by the RANs. A *p*‐value greater than 0.05 suggests no statistically significant difference between the two methods.

#### 2.2.3 Clinical applicability assessment

A two‐part reader study was designed to evaluate the clinical applicability of synthesized V/Q images from two complementary perspectives: (I) perceptual image acceptability for clinical review, and (II) illustrative diagnostic interpretation based on synthesized image pairs alone. The two parts of the reader study were conducted separately to avoid conflating perceptual image quality with diagnostic decision‐making.

For qualitative image quality assessment, four nuclear medicine physicians with varying levels of experience rated the quality of synthesized images using a five‐point Likert scale with predefined scoring anchors: 0 = dissimilar (completely unacceptable image quality); 1 = poor (widespread distortions); 2 = fair (obvious localized deviations); 3 = good (acceptable with room for further optimization); 4 = excellent (close to clinical images); 5 = perfect (indistinguishable from clinical images). Cases were presented in random order. During image quality scoring, the synthesized images and their corresponding real images were presented to the physicians simultaneously, with clear labels indicating which were synthesized. The primary objective was to manually assess the similarity between synthesized images and real images, thereby verifying the clinical utility of the model‐generated images.

Furthermore, to explore the diagnostic utility, an illustrative, hypothesis‐generating diagnostic reader study was conducted. Two nuclear medicine physicians were blinded to whether images were synthesized and were not provided with CT images or other clinical information. Only paired V/Q images were simultaneously shown, and cases were presented in randomized order. They independently assigned one of the predefined diagnostic categories (COPD, PE, lung cancer, pneumonia, or normal case), and their diagnoses were compared against the clinically adjudicated ground truth.

## 3 RESULTS

### 3.1 Qualitative results

**FIGURE 2:**
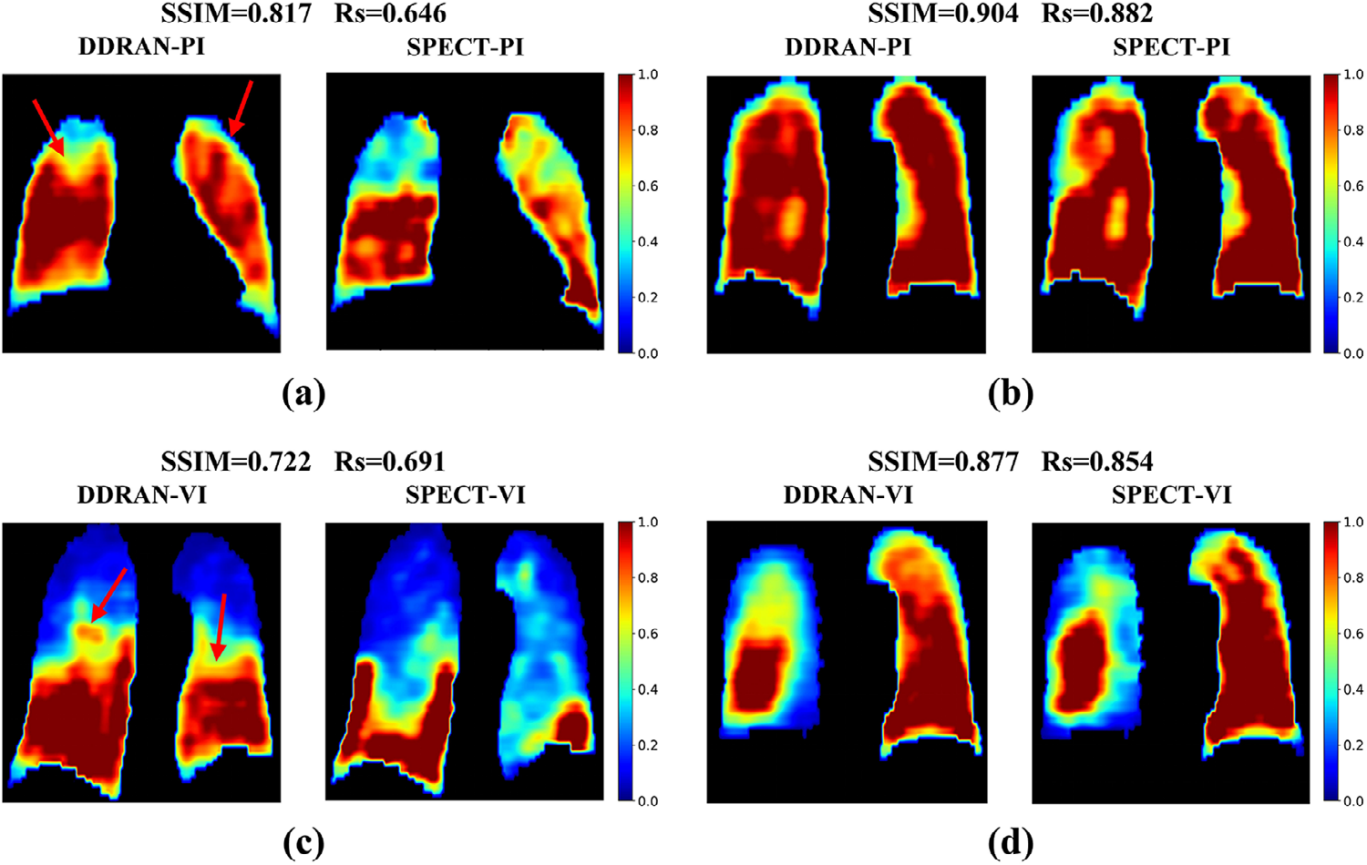
Qualitative results of synthesized lung function images. Four test cases are demonstrated in (a–d) from a view of the coronal plane. The upper row displays the synthesized lung perfusion images, while the bottom row shows the lung ventilation images. For the two well‐performing cases (b) and (d) shown above, our DDRAN model can generate lung functional images that resemble real SPECT images. For the two poorly performing cases (a) and (c), the synthesized lung functional images failed to capture the functional information in some low‐function regions, as indicated by the red arrows in the figures.

Figure 2 compares synthesized lung functional images and the corresponding SPECT images from the test dataset. The top row of Figure 2 presents two perfusion images, and all the examples are displayed in their coronal planes. For each subfigure, the left side shows the perfusion image generated by our DDRAN model, while the right side shows the corresponding real perfusion image. The annotations at the top of each subfigure represent the value of quantitative evaluation metrics for this test case. Similarly, the bottom row displays the qualitative results for ventilation images. Figure 2(a) and (c) exhibit scenarios with relatively subpar generation performance, whereas Figure 2(b) and (d) depict scenarios with markedly superior performance. It can be observed that the synthesized images closely resemble the ground truth in the superior cases, namely, Figure 2(b) and (d). For the inferior cases, the model exhibits errors where it predicts low‐function areas as high‐function ones, as indicated by the red arrows in the figures. This issue exists in both synthesized V/Q images and is comparatively more pronounced in the ventilation images.

**FIGURE 3:**
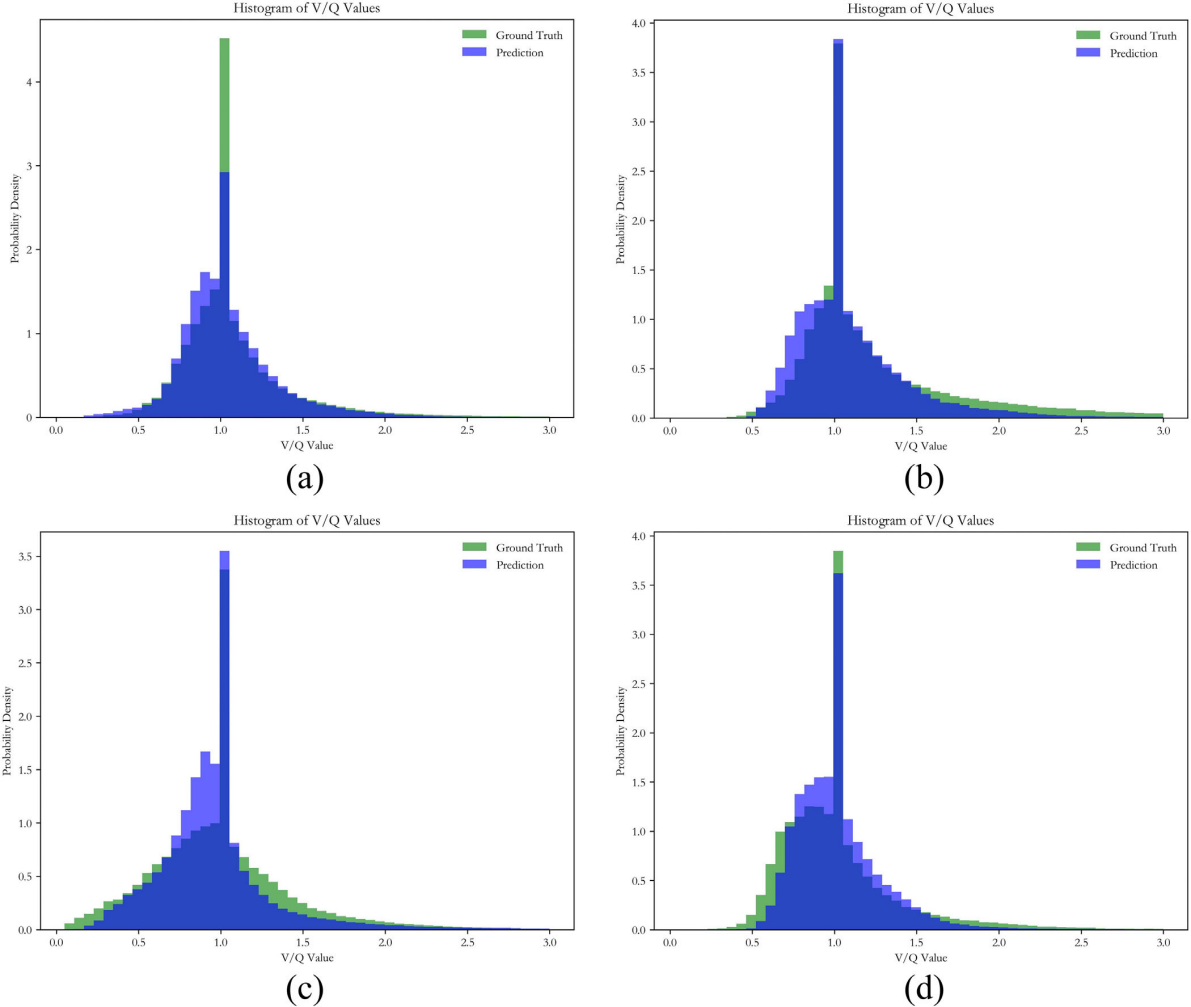
Histograms comparing the probability density distributions of ventilation‐perfusion ratios. Each panel presents a randomly selected test case. In each panel, the green one represents the ground truth and the blue one displays the prediction generated by our dual‐output model. Comparative analysis of the ground truth and predicted ventilation‐perfusion distributions reveals significant similarity in their probability density profiles.

Figure 3 demonstrates the comparative probability density histograms of ventilation‐perfusion ratios for four randomly selected cases from the test dataset, contrasting synthesized images against real images. These histograms provide an effective visual tool for evaluating how accurately our dual‐output model captures the complex physiological distribution of ventilation‐perfusion relationships in lung functional imaging. As shown in panels (a)‐(d) of Figure 3, the distributions of synthesized lung functional images generated by our dual‐output model exhibit substantial similarity in their overall morphology when compared with real images. Both distributions consistently display a primary peak located near the physiologically optimal ratio of 1.0. Furthermore, the dispersion patterns are similar, as evidenced by matching skewness and kurtosis characteristics on both sides of the central peak. This morphological agreement demonstrates that our dual‐output model successfully captures the underlying physiological distribution of ventilation‐perfusion ratios.

### 3.2 Quantitative results

**FIGURE 4:**
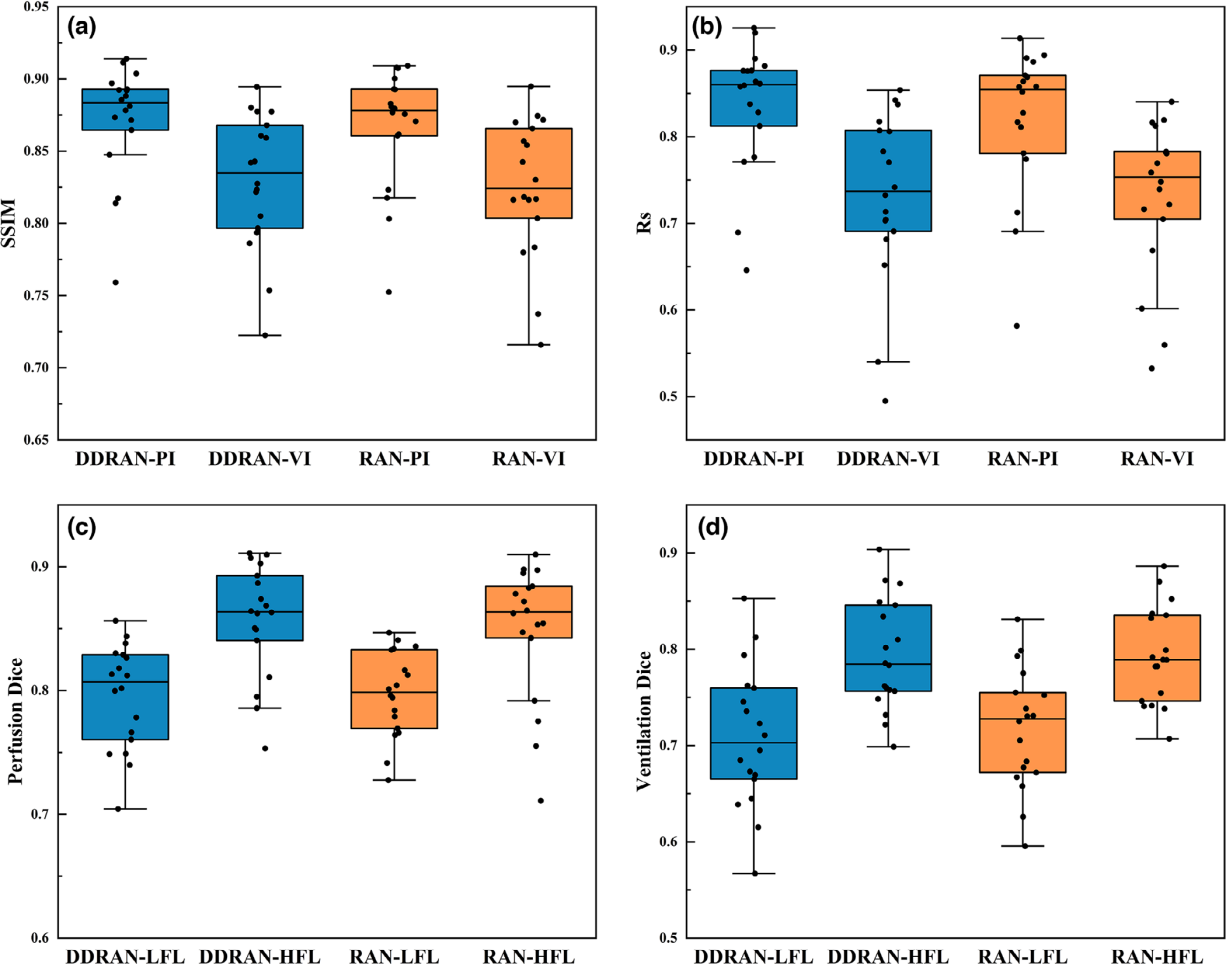
Statistical analysis of different evaluation metrics: (a) The comparison between the DDRAN model and RAN model in terms of SSIM. (b) Comparison of Rs. (c, d) Comparison of DSC for high‐function and low‐function regions, respectively. The DDRAN model demonstrated superior performance to the RAN model in terms of SSIM and Rs while achieving comparable DSC results.

Table 2 summarizes the mean SSIM, Rs, DSC, and *p*‐values of the DDRAN and RAN models. Figure 4 shows the box plots of the SSIM, Rs, and DSC to compare the results of the two models. The SSIM and Rs values of our DDRAN model are similar to those of each RAN model. The average SSIM values of our DDRAN model were 0.871 ± 0.038 for perfusion images and 0.830 ± 0.045 for ventilation images, and the Rs values were 0.836 ± 0.072 and 0.732 ± 0.096, respectively. The RAN models yielded SSIM values of 0.866 ± 0.041 and 0.825 ± 0.047, and Rs values of 0.819 ± 0.083 and 0.731 ± 0.086, respectively.

**TABLE 2:** The SSIM, Rs, and DSC values (mean ± SD) between the DDRAN and RAN models for the test set.

	Perfusion images			Ventilation images		
	DDRAN‐PI	RAN‐PI	*p*‐value	DDRAN‐VI	RAN‐VI	*p*‐value
SSIM	0.871 ± 0.038	0.866 ± 0.041	0.027	0.830 ± 0.045	0.825 ± 0.047	0.009
Rs	0.836 ± 0.072	0.819 ± 0.083	0.074	0.732 ± 0.096	0.731 ± 0.086	0.734
DSC for Low‐function area	0.795 ± 0.041	0.796 ± 0.034	0.966	0.708 ± 0.071	0.718 ± 0.061	0.196
DSC for High‐function area	0.857 ± 0.044	0.849 ± 0.053	0.074	0.793 ± 0.056	0.793 ± 0.049	0.799

Abbreviations: DSC, dice similarity coefficient; DDRAN, dual‐decoder residual attention network; PI, perfusion image; RAN, residual attention network; Rs, Spearman's similarity coefficient; SSIM, structural similarity index; VI, ventilation image.

For the DSC metric, the results of our DDRAN model are slightly lower than those of the RAN models. Our DDRAN model achieved DSC values of 0.795 ± 0.041 (perfusion) and 0.708 ± 0.071 (ventilation) in low‐function regions, and 0.857 ± 0.044 (perfusion) and 0.793 ± 0.056 (ventilation) in high‐function regions. In contrast, the DSC values of the RAN models were 0.796 ± 0.034 and 0.718 ± 0.061 in low‐function regions, and 0.849 ± 0.053 and 0.793 ± 0.049 in high‐function regions. Although our DDRAN model exhibits slightly lower values for some metrics, such as DSC, compared to the RAN models, all *p*‐values are greater than 0.05, signifying that the differences between the results of the two groups are not statistically significant. Furthermore, the *p*‐values for SSIM (for both ventilation and perfusion images) are less than 0.05, indicating a significant improvement in perceptual similarity.

**FIGURE 5:**
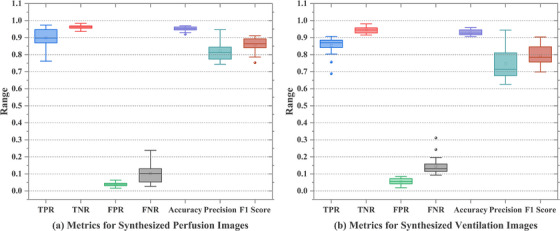
Box‐and‐whisker plots of evaluation metrics for synthesized perfusion and ventilation images. Subplot (a) illustrates the distribution of metrics (TPR, TNR, FPR, FNR, accuracy, precision, F1 Score) for synthesized perfusion images, while subplot (b) presents the corresponding distribution for synthesized ventilation images. The box plots depict the range, interquartile range, and central tendency of each metric, providing a visual summary of the models’ performance in classifying functional regions in synthesized perfusion and ventilation images.

Table 3 and Figure 5 present threshold‐based classification results for synthesized perfusion and ventilation images generated by the RAN and DDRAN models, using the same threshold as in the DSC analysis (values > 0.66 were mapped to the positive class; otherwise to the negative class). For synthesized perfusion images, the DDRAN model achieved higher sensitivity (TPR: 0.898 vs. 0.871) and lower FNR (0.102 vs. 0.129) than the RAN model, with comparable TNR (0.962 vs. 0.965) and accuracy (0.951 vs. 0.949). However, precision was marginally lower for DDRAN (0.823 vs. 0.830), and its F1 score was improved (0.857 vs. 0.849). For synthesized ventilation images, the DDRAN model similarly increased TPR (0.854 vs. 0.839) and reduced FNR (0.146 vs. 0.161), while maintaining similar TNR (0.945 vs. 0.949) and accuracy (0.930 vs. 0.931); precision remained slightly higher for the RAN model (0.758 vs. 0.747), and F1 scores were essentially equivalent (0.794 vs. 0.793). Collectively, these findings indicate that DDRAN operated at a more favorable operating point—delivering greater sensitivity to high‐function regions with minimal trade‐offs in specificity and overall accuracy—while keeping precision and F1 scores at competitive levels relative to the RAN models.

**TABLE 3:** Comparison of evaluation metrics for synthesized perfusion and ventilation images between the DDRAN and RAN models.

Evaluation Metrics	Perfusion images		Ventilation images	
	RAN	DDRAN	RAN	DDRAN
TPR	0.871	0.898	0.839	0.854
TNR	0.965	0.962	0.949	0.945
FPR	0.035	0.038	0.051	0.055
FNR	0.129	0.102	0.161	0.146
Accuracy	0.949	0.951	0.931	0.930
Precision	0.830	0.823	0.758	0.747
F1 Score	0.849	0.857	0.793	0.794

Abbreviations: DDRAN, dual‐decoder residual attention network; FNR, false negative rate; FPR, false positive rate; RAN, residual attention network; TNR, true negative rate; TPR, true positive rate.

### 3.3 Reader study results

#### 3.3.1 Perceptual image acceptability results

**FIGURE 6:**
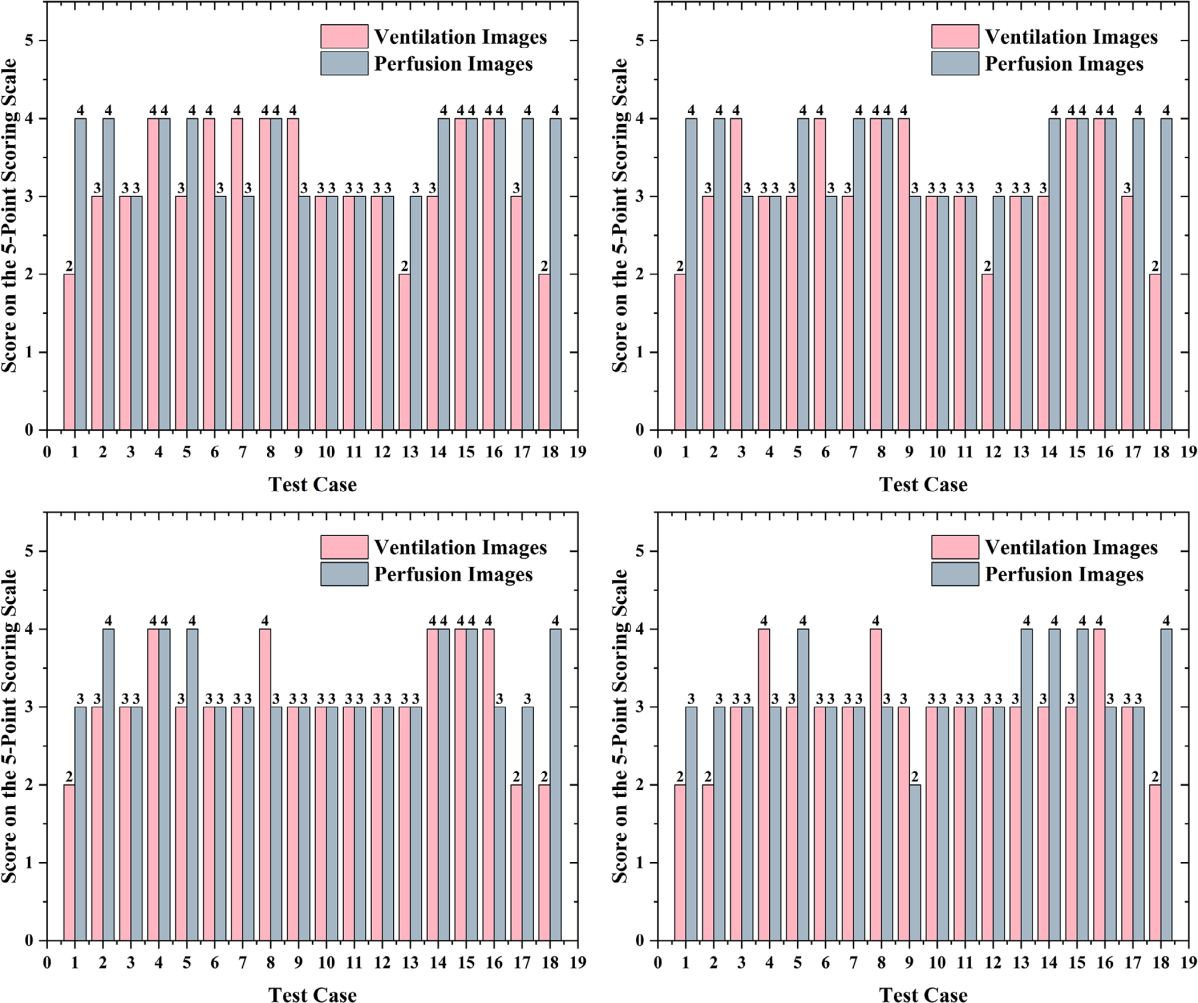
Reader study scores of synthesized perfusion and ventilation images across four nuclear medicine physicians with varying experience levels. Each subplot depicts the 5‐point scale scores (0 = completely unacceptable image quality, 5 = image quality indistinguishable from clinically acquired images) for all test cases, with pink bars for ventilation images and blue bars for perfusion images. The subplots from top‐left to bottom‐right correspond to a resident, two junior physicians, and a senior physician, respectively. These scores reflect the clinical acceptability of synthesized functional images as evaluated by physicians with different levels of expertise.

We conducted a reader study with four nuclear medicine physicians at different experience levels to qualitatively evaluate perceptual image acceptability. Each physician rated the quality of synthesized perfusion and ventilation images with a 5‐point Likert scale, and the results are shown in Figure 6. In the four subplots, the panels from top left to bottom right correspond to a resident, two junior physicians, and a senior physician, respectively. Each panel displays scores for all the test cases. Overall, the synthesized images were rated favorably across most cases and readers, indicating broad acceptance across experience levels. Notably, the senior physician (bottom‐right panel) and the other readers assigned scores that support the potential clinical utility, although some variability was observed across cases and between image types. Taken together, these findings provide evidence for the clinical acceptability of the synthesized perfusion and ventilation images.

#### 3.3.2 Illustrative diagnosis results

**FIGURE 7:**
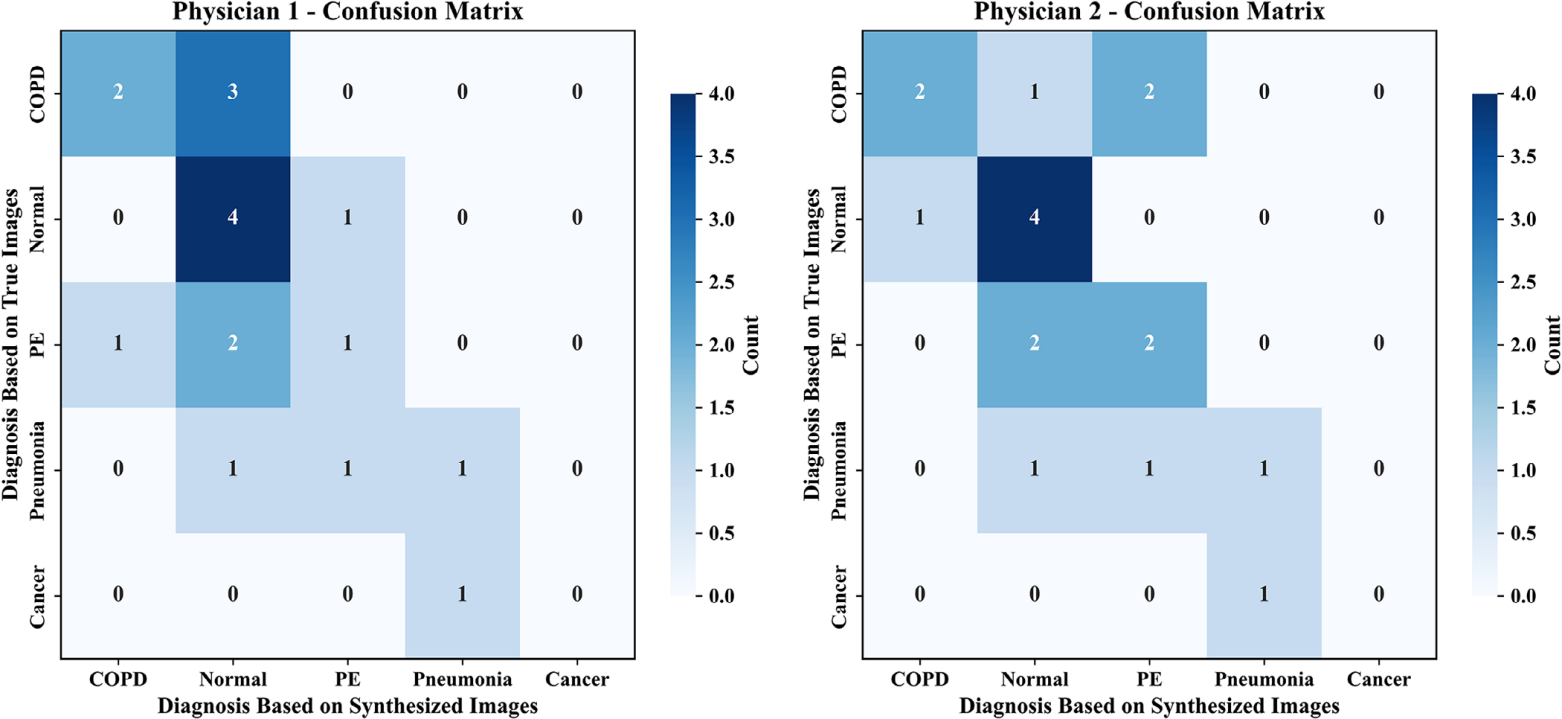
Confusion matrices of diagnostic results by two nuclear medicine physicians using paired synthesized functional images. The left subplot depicts the confusion matrix for physician 1, and the right subplot depicts the confusion matrix for physician 2. Rows represent the ground truth diagnoses (COPD, normal case, PE, pneumonia, lung cancer), while columns denote the diagnoses based on synthesized functional images. The color scale indicates the number of diagnoses, with darker shades corresponding to higher frequencies. These matrices illustrate common diagnostic confusions when relying solely on synthesized perfusion and ventilation images. Given the small number of cases per disease category and only two readers, the analysis is illustrative and hypothesis‐generating.

To illustrate diagnostic interpretation patterns when relying solely on synthesized functional images, we summarized the two readers’ categorical decisions using confusion matrices relative to the clinically adjudicated reference labels (COPD, normal case, PE, pneumonia, cancer), as shown in Figure 7. For physician 1, COPD was correctly identified in 2 of 5 cases (the remaining three were mainly labeled normal case); Normal case in 4 of 5 (1 labeled PE); PE in 2 of 4 (2 assigned to other categories); Pneumonia in 1 of 3 (2 assigned to other categories). For physician 2, COPD was correct in 2 of 5 (with one labeled normal case and 2 labeled PE); Normal case in 4 of 5 (1 labeled COPD); PE in 2 of 4 (2 assigned to other categories); Pneumonia in 1 of 3 (2 assigned to other categories). Overall, most cross‐confusions occurred among COPD, normal case, and PE, whereas both readers misclassified the single lung cancer case as pneumonia. These patterns qualitatively delineate the core dimensions of diagnostic ambiguity when relying solely on synthesized images, and provide illustrative and hypothesis‐generating evidence for their potential diagnostic utility.

## 4 DISCUSSION

In this study, we developed a DDRAN model capable of simultaneously generating perfusion and ventilation images from 3D CT. Our model achieved moderate‐to‐high performance in synthesizing perfusion and ventilation images and exhibited no statistically significant difference compared to the single‐decoder models. To our knowledge, this represents the first study to simultaneously generate both perfusion and ventilation images directly from 3D CT within a single framework.

Quantitative results are summarized in Table 2. Overall, DDRAN achieved moderate‐to‐high agreement with reference SPECT for both perfusion and ventilation synthesis, and its performance was broadly comparable to that of the single‐decoder baselines across voxel‐wise and region‐wise metrics. The Wilcoxon signed‐rank test indicated a small but statistically significant difference in SSIM between DDRAN and the single‐decoder models. In contrast, no statistically significant differences were observed in the other metrics. However, given the small absolute SSIM differences, the clinical significance of this improvement warrants further validation. A consolidated comparison with representative prior work is provided in Table 4. Compared with prior CT‐to‐perfusion or CT‐to‐ventilation studies, our framework enables paired perfusion–ventilation assessment from a single 3D CT in one forward pass, providing consistent voxel‐wise pairing while maintaining comparable accuracy.

**TABLE 4:** Comparison of different methods for lung functional image synthesis.

Methods	Networks	Input images	Output modalities	Dataset size	SSIM	Rs	DSC (LFA)	DSC (MFA)	DSC (HFA)
Ren et al.^37^	3D ResU‐Net	CT	Perfusion	73	0.76	0.67	0.65	–	0.82
Gu et al.^41^	3D Unet	CT+PET	Perfusion	53	0.93	0.78	0.82	–	0.77
Porter et al.^54^	3D ResU‐Net	4D CT	Perfusion	32	–	0.70	0.80		
Kajikawa et al.^39^	3D U‐Net	CT	Ventilation	71	–	0.76	0.69	0.51	0.75
Our	3D ResU‐Net	CT	Perfusion	98	0.87	0.84	0.80	–	0.86
			Ventilation	98	0.83	0.73	0.71	–	0.79

Abbreviations: CT, computed tomography; DSC, dice similarity coefficient; 4D, four‐dimensional; HFA, high‐function area; LFA, low‐function area; MFA, moderate ‐function area; PET, positron emission tomography; ResU‐Net, residual U‐Net; Rs, Spearman's rank correlation coefficient; SSIM, structural similarity index; 3D, three‐dimensional.

The threshold‐based classification analysis (as shown in Table 3 and Figure 5) shows that the DDRAN model operates at a more favorable point than the single‐decoder models—achieving higher sensitivity and lower false‐negative rates for both perfusion and ventilation while maintaining comparable specificity, accuracy, and F1 score. Interpreted clinically, the reduction in false negatives is desirable because it decreases the risk of misclassifying high‐function regions as low‐function, which could otherwise lead to unnecessarily permissive dosing of functionally preserved lung in FLART. The small differences observed in specificity (and therefore FPR) represent a modest trade‐off that is acceptable in scenarios prioritizing protection of high‐function regions. Overall, the results support equivalent performance alongside added efficiency and workflow benefits.

The reader study provided further evidence for the clinical applicability of the method. Among physicians with different experience levels, the synthesized images received generally favorable quality ratings on the 5‐point Likert scale, indicating broad perceptual acceptance. Although inter‐case variability was evident, the overall distribution of scores suggests that the synthesized perfusion and ventilation images are of a certain quality for clinical review across experience strata. The diagnostic reader study provided preliminary insight into how much disease‐specific information is retained in synthesized image pairs when CT and clinical context are withheld. Confusion matrices derived from two experts who relied solely on the synthesized functional images revealed that most cross‐confusions clustered among COPD, normal, and PE, delineating the principal axes of diagnostic ambiguity. The main driver of this pattern appears to be the model's occasional overestimation of perfusion and underestimation of ventilation. Consequently, patients with PE tend to be misclassified as normal case (owing to overestimated perfusion) or as COPD (owing to underestimated ventilation). Similarly, pneumonia may be misclassified as normal case or PE. For the single lung cancer case, functional images alone were insufficient to distinguish it from pneumonia because their functional signatures were highly similar, and combination with CT images was required for a more precise diagnosis. This diagnostic reading is intended to be illustrative. Further larger, prospectively designed multi‐reader studies would be valuable to rigorously evaluate diagnostic performance.

Collectively, these findings indicate that the dual‐output DDRAN model delivers both performance and systems‐level benefits. Despite producing two modalities simultaneously, the DDRAN model achieves a more favorable operating point (higher sensitivity with minimal specificity trade‐offs) and, by sharing a single encoder for perfusion and ventilation, materially reduces the number of parameters and computational burden relative to maintaining two separate single‐decoder models. In practice, generating both maps in a single forward pass cuts training time, inference time, and memory footprint, simplifies deployment and version control (one model instead of two), and guarantees voxel‐wise co‐registration between perfusion and ventilation images without post‐hoc processing. The shared features in the encoder also encourage cross‐modality consistency, which aligns with the observed gains in sensitivity and the favorable 5‐point reader ratings. In aggregate, these advantages enhance the practical value and scalability of multimodal diagnosis, enabling faster turnaround on GPUs while maintaining (and in some cases improving) clinically relevant performance.

From a clinical utility standpoint, our study also exhibits promising potential. Our model is highly beneficial for emergency patients who urgently need information about their lung function. Upon obtaining the patient's CT images, our model can predict their ventilation and perfusion images within seconds, eliminating the need for the time‐consuming process of V/Q SPECT imaging. Even if our model's performance may not be exceptional, it can still provide a rapid reference for physicians. Another potential application of our model lies in FLART. Currently, SPECT imaging is not incorporated into the standard treatment protocol for lung cancer patients undergoing radiotherapy. When designing radiotherapy plans for lung cancer patients, we assume a uniform lung function distribution, considering all functional regions within the lungs as equivalent during the optimization of treatment planning. However, in reality, the functional distribution within the lungs is inhomogeneous. Previous studies^55^ have reported that, in comparison to the dosimetric parameters based on the entire lung in traditional radiotherapy, the dosimetric parameters of high‐ventilation functional regions of the lungs exhibit a stronger correlation with the occurrence of radiation pneumonitis following photon or proton radiotherapy. Medical physicists can initially apply our model to derive lung ventilation and perfusion images from CT during radiotherapy planning, providing a thorough understanding of the patient's lung function distribution. Consequently, a function‐guided optimization strategy becomes feasible: doses are focused on low‐function regions to spare high‐function lung, all while maintaining target coverage. This methodology holds the potential to decrease the incidence and severity of RILI in lung cancer patients after radiotherapy, ultimately improving their prognosis.

Although our study demonstrates the feasibility of simultaneously synthesizing both perfusion and ventilation images, several limitations should be acknowledged. First, we chose the same loss function weights for ventilation and perfusion images during training and validation. However, our results showed that the values of all evaluation metrics for ventilation images were lower (SSIM ∼5%, Rs ∼12%, DSC _LFL_ ∼11%, DSC _HFL_ ∼7%) than those of perfusion images. A potential cause for the suboptimal agreement in the probability density distributions (Figure 3) lies in the acquisition‐related limitations of ventilation imaging. Specifically, inadequate tracer inhalation leads to low count rates, yielding reconstructed images of inferior spatial resolution that negatively impact both model training and testing outcomes. On the other hand, our preprocessing pipeline may not adequately compensate for these inherent deficiencies; applying a uniform normalization threshold to both perfusion and ventilation images could be suboptimal given their divergent image quality baselines. These factors likely contribute to the inferior performance of synthesized ventilation images. Further investigation is needed to understand the impact of these preprocessing steps and parameters on model performance.

Secondly, in cases of inferior performance (Figure 2), low‐function regions were frequently misclassified as high‐function regions—a trend consistent with prior studies.^37^, ^39^ This bias likely stems from the class imbalance between low‐ and high‐function regions in the training data. Future work should focus on enhancing the network's sensitivity to underrepresented low‐function regions. Finally, the current dataset, though valuable, remains modest in scale and diversity. Additionally, the retrospective design lacks prospective validation, and external validation across multiple institutions was not performed. To address these considerations, we will continue to accrue multi‐center patient data, further diversify and expand the dataset in terms of disease subtypes and sample size, and aim to optimize the model for improved performance. Furthermore, we will integrate the synthesized functional images into radiotherapy planning processes to evaluate dose‐volume trade‐offs and correlate them with clinical endpoints (e.g., RILI incidence), thereby substantiating their clinical value.

## 5 CONCLUSIONS

This study establishes a deep learning framework (DDRAN) that simultaneously generates ventilation and perfusion images from routine 3D CT, reducing redundancy in deployment and ensuring voxel‐wise co‐registration between perfusion and ventilation outputs. Preliminary findings indicate that DDRAN exhibits moderate‐to‐high performance in both structural and functional aspects, showcasing its proficiency in concurrent prediction of perfusion and ventilation images. The synthesized images show promise for precise lung disease diagnosis and could potentially be utilized in FLART to improve the prognosis of lung cancer patients. Future efforts will prioritize external validation across diverse, multi‐center cohorts to solidify its clinical robustness and generalizability.

## AUTHOR CONTRIBUTIONS

*Conceptualization*: Ruijie Yang, Li‐Sheng Geng, Xi Liu, David Huang, and Jing Cai. *Methodology*: Xi Liu, Haoze Li, Meng Wang, Li‐Sheng Geng, Tianyu Xiong, Meixin Zhao, and Weifang Zhang. *Data curation*: Meng Wang, Xi Liu, Meixin Zhao, and Weifang Zhang. *Analysis*: Meng Wang, Xi Liu, Haoze Li, and Tianyu Xiong. *Project administration and funding acquisition*: Ruijie Yang, Li‐Sheng Geng, Weifang Zhang, and Meng Wang. *Writing—original draft*: Xi Liu, Meng Wang, and Haoze Li. *Writing—review and editing*: All authors.

## CONFLICT OF INTEREST STATEMENT

The authors have no relevant conflicts of interest to disclose.

## ETHICS STATEMENT

This study was conducted in accordance with the Declaration of Helsinki, and approved by the Peking University Third Hospital Medical Science Research Ethics Committee (protocol code IRB00006761‐M2024673).

## Data Availability

The datasets generated and/or analyzed in this study are not publicly available due to patient privacy and ethical restrictions but are available from the corresponding author on reasonable request.
